# Circulating biomarkers and progression of idiopathic pulmonary fibrosis: data from the INMARK trial

**DOI:** 10.1183/23120541.00335-2023

**Published:** 2024-07-22

**Authors:** Toby M. Maher, R. Gisli Jenkins, Vincent Cottin, Yasuhiko Nishioka, Imre Noth, Moisés Selman, Jin Woo Song, Carina Ittrich, Claudia Diefenbach, Susanne Stowasser, Eric S. White

**Affiliations:** 1 National Heart and Lung Institute, Imperial College London, London, UK; 2 Keck School of Medicine, University of Southern California, Los Angeles, CA, USA; 3 National Reference Coordinating Centre for Rare Pulmonary Diseases, Louis Pradel Hospital, Hospices Civils de Lyon, Claude Bernard University Lyon 1, UMR754, Lyon, France; 4 Graduate School of Biomedical Sciences, Tokushima University, Tokushima, Japan; 5 Division of Pulmonary and Critical Care Medicine, University of Virginia, Charlottesville, VA, USA; 6 Instituto Nacional de Enfermedades Respiratorias “Ismael Cosio Villegas”, Mexico City, Mexico; 7 Department of Pulmonary and Critical Care Medicine, Asan Medical Center, University of Ulsan College of Medicine, Seoul, South Korea; 8 Boehringer Ingelheim Pharma GmbH & Co. KG, Biberach an der Riss, Germany; 9 Boehringer Ingelheim International GmbH, Ingelheim am Rhein, Germany; 10 Boehringer Ingelheim Pharmaceuticals, Inc., Ridgefield, CT, USA

## Abstract

**Background:**

We used data from the INMARK trial to investigate associations between circulating biomarkers of extracellular matrix (ECM) turnover, inflammation and epithelial dysfunction and disease progression in subjects with idiopathic pulmonary fibrosis (IPF).

**Methods:**

Subjects with IPF and forced vital capacity (FVC) ≥80% predicted were randomised 1:2 to receive nintedanib 150 mg twice daily or placebo for 12 weeks followed by open-label nintedanib for 40 weeks. Associations between baseline biomarker levels and the proportion of subjects with disease progression (decline in FVC ≥10% predicted or death) over 52 weeks were assessed in subjects randomised to placebo using logistic regression. Associations between baseline demographic/clinical characteristics and biomarker levels and disease progression over 52 weeks were analysed using multivariate models.

**Results:**

Of 230 subjects who received placebo for 12 weeks then open-label nintedanib for 40 weeks, 70 (30.4%) had disease progression over 52 weeks. Baseline levels of CRPM (C-reactive protein (CRP) degraded by matrix metalloproteinase (MMP)-1/8), C3M (collagen 3 degraded by MMP-9), CRP, KL-6 (Krebs von den Lungen-6) and SP-D (surfactant protein D) were not significantly associated with disease progression over 52 weeks in analyses corrected for multiple comparisons. In models including only baseline demographic/clinical characteristics, 61.2–64.2% of subjects were correctly classified as having or not having disease progression over 52 weeks. When both demographic/clinical characteristics and biomarker levels were included in the models, 50.0–64.5% of the test set were correctly classified.

**Conclusions:**

Among subjects with IPF and preserved FVC, multivariate models based on demographic/clinical characteristics and biomarker levels at baseline did not provide an accurate prediction of which patients would progress.

## Shareable abstract

**In patients with idiopathic pulmonary fibrosis, multivariate models based on demographic/clinical characteristics and circulating biomarker levels at baseline did not provide an accurate prediction of disease progression over 52 weeks** https://bit.ly/3THYJ2C

## Introduction

Idiopathic pulmonary fibrosis (IPF) is a progressive fibrosing interstitial lung disease associated with high mortality [1]. The pathogenesis of IPF is believed to involve activation of epithelial cells in response to injury, which leads to fibroblast migration and proliferation and the differentiation of fibroblasts into myofibroblasts. Extracellular matrix (ECM) components secreted by myofibroblasts accumulate and lead to aberrant remodelling of the lung architecture and the pathology characteristic of fibrosis [2]. IPF is always progressive but varies in its rate of progression. A number of circulating biomarkers, including those associated with ECM turnover, epithelial injury and inflammation, have been associated with disease progression in subjects with IPF [3–11], but their clinical utility remains to be established.

The INMARK trial investigated circulating biomarkers as predictors of disease progression, and the effect of nintedanib on changes in these biomarkers, in subjects with IPF and preserved forced vital capacity (FVC) [12]. The primary results showed that treatment with nintedanib for 12 weeks did not significantly affect the rate of change in CRPM (C-reactive protein (CRP) degraded by matrix metalloproteinase (MMP)-1/8) compared with placebo [12]. Among subjects who received placebo for 12 weeks followed by open-label nintedanib for 40 weeks, there was no significant association between the rate of change in CRPM over 12 weeks and disease progression over 52 weeks; however, rising levels of CRPM over 12 weeks were associated with disease progression over 52 weeks [12]. In these analyses, we investigated associations between circulating biomarkers of ECM turnover, inflammation and epithelial dysfunction at baseline and over 12 weeks of treatment, changes in FVC over 12 weeks, and disease progression over 52 weeks in the INMARK trial.

## Methods

### Trial design

The design of the INMARK trial has been published [12]. Briefly, the trial enrolled subjects with IPF diagnosed according to the 2011 international guidelines [13] within the previous 3 years and FVC ≥80% predicted. Subjects were randomised 1:2 to receive nintedanib or placebo for 12 weeks, followed by an open-label period in which all subjects received nintedanib for 40 weeks. These analyses were conducted in subjects who were randomised to receive placebo and who received at least one dose of trial medication.

The INMARK trial was conducted in accordance with the principles of the Declaration of Helsinki and the Harmonized Tripartite Guideline for Good Clinical Practice from the International Conference on Harmonization and was approved by local authorities. The clinical protocol was approved by an independent ethics committee or institutional review board at each participating centre. All patients provided written informed consent before study entry.

### Biomarkers

The following biomarkers were assessed as markers of ECM turnover: CRPM, C1M (collagen 1 degraded by MMP-2/9/13), C3M (collagen 3 degraded by MMP-9), BGM (biglycan degraded by MMP), C3A (collagen 3 degraded by ADAMTS-1/4/8), C5M (collagen 5 degraded by MMP-2/9), C6M (collagen 6 degraded by MMP-2/9), VICM (citrullinated vimentin degraded by MMP-2/8), pro-C3 (N-terminal propeptide of type III collagen), pro-C6 (N-terminal propeptide of type VI collagen), LOXL2 (lysyl oxidase-like 2) and EL-NE (neutrophil-specific elastin fragments). KL-6 (Krebs von den Lungen-6), SP-D (surfactant protein D), CA-125 and CA19-9 were assessed as markers of epithelial injury. CRP and ICAM-1 (intercellular adhesion molecule-1) were assessed as markers of inflammation.

### Sample preparation and analysis

For serum samples, blood was collected with anticoagulant-free, gel-containing serum separation tubes and left to clot at room temperature for ∼1 h. The serum was separated by centrifugation and aliquoted before freezing. For plasma samples, blood was collected with K2 EDTA plasma tubes and inverted 8–10 times. The plasma was separated by centrifugation and aliquoted before freezing. Samples were shipped from a central laboratory to the sponsor or a contractor for analysis. Serum concentrations of each biomarker of ECM turnover were measured using ELISA [14]. Plasma concentrations of KL-6 and SP-D were measured using commercially available ELISA methods with minor adaptations (KL-6: Sanko Junyaku/EIDIA; SP-D: BioVendor). Serum concentrations of CA-125 were measured using an electrochemiluminescence immunoassay (Beckman DxI 800). Serum concentrations of LOXL2 were measured using ELISA (Nordic Bioscience). Plasma concentrations of ICAM-1 and serum concentrations of CA19-9 were measured using electrochemiluminescence immunoassays (ICAM-1: Merck Sharp & Dohme; CA19-9: Roche Cobas e-601).

### Analyses

Biomarker data were not normally distributed and were log_10_ transformed (or negative reciprocal root transformed for C1M) prior to analysis. Relationships between biomarker levels at baseline and the adjusted rate of decline in FVC (mL) over 12 weeks were assessed by analysing the rate of decline in FVC per unit increase in log value of each biomarker. The rate of decline in FVC (mL) over 12 weeks was analysed using a random coefficient regression model (with random slopes and intercepts) including fixed categorical effects of sex, age, height; fixed continuous effects of baseline FVC (mL), and baseline biomarker value and batch number (only for C1M, EL-NE and pro-C6) as well as baseline FVC×time, baseline biomarker×time interactions and batch number×time (only for C1M, EL-NE and pro-C6). Within-patient errors were modelled by an unstructured variance–covariance matrix. p-values were corrected for multiple comparisons using the Benjamini–Hochberg method [15] to control the false discovery rate (FDR) at 5%.

Correlations between changes from baseline in each biomarker at week 4 and changes from baseline in FVC % predicted at week 12 were assessed using the Spearman correlation coefficient (ρ), with Fisher's z-transformation and bias correction. Associations between each biomarker and the proportion of subjects with disease progression (decline in FVC ≥10% predicted or death) over 52 weeks were assessed based on: 1) baseline biomarker levels and 2) baseline biomarker levels plus the rate of change in the biomarker over the first 12 weeks. The rate of change in each biomarker was analysed based on the continuous monthly rate of change and based on rising *versus* stable or falling levels. Associations were analysed using logistic regression with the baseline value of the biomarker as a linear covariate. Analyses including the rate of change in each biomarker had an additional term for continuous monthly rate of change or rising *versus* stable or falling levels.

Associations were assessed between baseline FVC % predicted, baseline diffusing capacity of the lung for carbon monoxide (*D*_LCO_) % predicted and the baseline value of the biomarker and disease progression over 52 weeks based on logistic regression. The covariates included in the model were the baseline values of FVC % predicted, *D*_LCO_ % predicted and each biomarker, all assessed as continuous covariates, plus batch for C1M, EL-NE and pro-C6 (which were analysed in two batches at baseline). p-values based on a log-rank test compared models with and without the baseline value of the biomarker included as a covariate.

Associations between baseline demographic/clinical characteristics (age, sex, body mass index, race, FVC % predicted and *D*_LCO_ % predicted) and the baseline value of the biomarker and disease progression over 52 weeks were analysed using multivariate LASSO (least absolute shrinkage and selection operator) with stability selection across 100 random subsamples of size 0.5×size of the dataset [16] and random forest regression models. Five sets of demographic/clinical characteristics and biomarkers were chosen. The proportions of subjects correctly classified as having or as not having disease progression over 52 weeks were assessed in a training set (approximately two-thirds of the subjects) and then in a test set (approximately one-third of the subjects). Mean coefficients across the 100 repetitions of the LASSO or importance values of the random forest regression models for the demographic/clinical characteristics and biomarkers selected in each of the five sets were calculated. Demographic/clinical characteristics and biomarkers with selection frequency ≥25% in the LASSO model with stability selection or with importance values ≥2.5 in the random forest model are presented.

## Results

### Subjects

A total of 230 subjects received placebo in the double-blind period. Their baseline characteristics have been published [12]. In summary, most were male (73.5%), White (62.6%) and ex-smokers (68.7%). At baseline, mean±sd age was 70.2±7.2 years, FVC was 98.0±12.6% predicted and *D*_LCO_ was 65.5±21.2% predicted.

### Relationship between biomarker levels at baseline and rate of decline in FVC over 12 weeks

There was no significant relationship between biomarker levels at baseline and the rate of decline in FVC over 12 weeks (table 1). For the biomarkers other than C1M, rates of decline in FVC over 12 weeks per unit increase in log value of the biomarker at baseline ranged from −9.8 to 21.9 mL over 12 weeks.

**TABLE 1:** Relationships between baseline biomarker levels and the rate of decline in forced vital capacity (FVC) over 12 weeks

Biomarker	Subjects (n)	Estimate (95% CI) for relationship between baseline biomarker level and rate of FVC decline over 12 weeks^#^	p-value	FDR-corrected p-value
**CRPM, ng·mL^−1^**	228	21.9 (−14.3–58.0)	0.23	1.00
**C1M, ng·mL^−1^**	227	191.7 (−555.0–938.4)	0.61	1.00
**C3M, ng·mL^−1^**	228	8.8 (−28.6–46.1)	0.64	1.00
**BGM, ng·mL^−1^**	226	−8.2 (−36.9–20.5)	0.57	1.00
**C3A, ng·mL^−1^**	228	0.0 (−25.1–25.2)	1.00	1.00
**C5M, ng·mL^−1^**	226	−2.6 (−44.1–38.9)	0.90	1.00
**C6M, ng·mL^−1^**	225	−9.8 (−38.2–18.5)	0.49	1.00
**VICM, ng·mL^−1^**	228	6.1 (−23.3–35.4)	0.68	1.00
**Pro-C3, ng·mL^−1^**	220	6.1 (−30.9–43.1)	0.75	1.00
**Pro-C6, ng·mL^−1^**	218	2.4 (−61.7–66.4)	0.94	1.00
**LOXL2, ng·mL^−1^**	170	−5.6 (−27.5–16.3)	0.61	1.00
**EL-NE, ng·mL^−1^**	226	4.2 (−35.7–44.1)	0.84	1.00
**KL-6, U·mL^−1^**	229	−0.4 (−15.5–14.8)	0.96	1.00
**SP-D, ng·mL^−1^**	228	6.3 (−10.0–22.6)	0.45	1.00
**CA-125, U·mL^−1^**	154	5.8 (−25.0–36.6)	0.71	1.00
**CA19-9, U·mL^−1^**	141	11.6 (−6.6–29.7)	0.21	1.00
**CRP, mg·L^−1^**	221	−9.8 (−32.5–12.9)	0.40	1.00
**ICAM-1, ng·mL^−1^**	228	5.7 (−11.0–22.4)	0.50	1.00

See Methods for details of biomarkers. FDR: false discovery rate. ^#^: estimates represent the rate of decline in FVC (mL) over 12 weeks per unit increase in log (or negative reciprocal root transformed for C1M) value of the biomarker at baseline; negative estimates indicate a greater rate of decline in FVC in patients with a higher biomarker value at baseline.

### Correlations between changes in biomarkers at week 4 and changes in FVC % predicted at week 12

No or weak correlations were observed between changes in biomarker levels at week 4 and changes in FVC % predicted at week 12 (supplementary table E1). Spearman correlation coefficients ranged from −0.01 to −0.20 and from 0.01 to 0.11.

### Associations between biomarker levels and disease progression over 52 weeks

Over 52 weeks, 70 subjects (30.4%) had disease progression. In analyses including the baseline biomarker level as a covariate, baseline levels of CRPM (OR 1.84 (95% CI 1.04–3.25)), C3M (OR 2.04 (95% CI 1.05–3.94)), CRP (OR 1.21 (95% CI 1.01–1.45)), KL-6 (OR 1.43 (95% CI 1.05–1.95)) and SP-D (OR 1.44 (95% CI 1.06–1.96)) were significantly associated with disease progression in uncorrected analyses, but not in analyses corrected for multiple comparisons (table 2). There were no significant associations between baseline biomarker levels and disease progression over 52 weeks in FDR-corrected analyses adjusted for baseline FVC % predicted and *D*_LCO_ % predicted (supplementary table E2). In FDR-corrected analyses including both the baseline level and the continuous rate of change of the biomarker over 12 weeks as covariates, there were no significant associations between baseline levels of biomarkers and disease progression (table 3). Adding the rate of change over 12 weeks as a covariate had no influence on the associations between baseline biomarker levels and disease progression. There were no significant associations between rising *versus* stable or falling levels of biomarkers over 12 weeks and disease progression over 52 weeks in FDR-corrected analyses including baseline levels and rising *versus* stable or falling levels over 12 weeks as covariates (supplementary table E3).

**TABLE 2:** Associations between baseline levels of biomarkers and disease progression over 52 weeks

Biomarker	OR (95% CI) for disease progression for baseline level
**CRPM, ng·mL^−1^**	1.84 (1.04–3.25)^#^
**C1M, ng·mL^−1^**	27.53 (0.10– >999.99)
**C3M, ng·mL^−1^**	2.04 (1.05–3.94)^#^
**BGM, ng·mL^−1^**	1.21 (0.88–1.66)
**C3A, ng·mL^−1^**	1.20 (0.70–2.06)
**C5M, ng·mL^−1^**	0.89 (0.58–1.36)
**C6M, ng·mL^−1^**	1.13 (0.80–1.59)
**VICM, ng·mL^−1^**	0.98 (0.77–1.24)
**Pro-C3, ng·mL^−1^**	1.05 (0.61–1.80)
**Pro-C6, ng·mL^−1^**	0.97 (0.62–1.53)
**LOXL2, ng·mL^−1^**	1.11 (0.81–1.53)
**EL-NE, ng·mL^−1^**	0.85 (0.63–1.13)
**KL-6, U·mL^−1^**	1.43 (1.05–1.95)^#^
**SP-D, ng·mL^−1^**	1.44 (1.06–1.96)^#^
**CA-125, U·mL^−1^**	0.98 (0.66–1.46)
**CA19-9, U·mL^−1^**	1.07 (0.88–1.31)
**CRP, mg·L^−1^**	1.21 (1.01–1.45)^#^
**ICAM-1, ng·mL^−1^**	1.53 (0.74–3.18)

See Methods for details of biomarkers. ^#^: p<0.05 in uncorrected analyses, p>0.05 in false discovery rate-corrected analyses.

**TABLE 3:** Associations between baseline levels plus continuous rate of change in biomarkers over 12 weeks and disease progression over 52 weeks

Biomarker	OR (95% CI) for disease progression for baseline level
**CRPM, ng·mL^−1^**	2.00 (1.10–3.65)^#^
**C1M, ng·mL^−1^**	26.39 (0.005– >999.99)
**C3M, ng·mL^−1^**	2.65 (1.07–6.58)^#^
**BGM, ng·mL^−1^**	1.50 (0.95–2.37)
**C3A, ng·mL^−1^**	1.22 (0.70–2.10)
**C5M, ng·mL^−1^**	0.90 (0.59–1.38)
**C6M, ng·mL^−1^**	1.14 (0.76–1.70)
**VICM, ng·mL^−1^**	0.99 (0.78–1.25)
**Pro-C3, ng·mL^−1^**	1.04 (0.60–1.80)
**Pro-C6, ng·mL^−1^**	0.83 (0.49–1.39)
**LOXL2, ng·mL^−1^**	1.18 (0.81–1.72)
**EL-NE, ng·mL^−1^**	0.93 (0.67–1.30)
**KL-6, U·mL^−1^**	1.47 (1.07–2.01)^#^
**SP-D, ng·mL^−1^**	1.42 (1.04–1.94)^#^
**CA-125, U·mL^−1^**	0.97 (0.66–1.44)
**CA19-9^¶^, U·mL^−1^**	1.07 (0.88–1.31)
**CRP^¶^, mg·L^−1^**	1.21 (1.01–1.45)^#^
**ICAM-1, ng·mL^−1^**	1.55 (0.75–3.22)

See Methods for details of biomarkers. ^#^: p<0.05 in uncorrected analyses, p>0.05 in false discovery rate-corrected analyses; ^¶^: model includes only baseline levels of the biomarker since no individual slopes could be estimated in the placebo group.

Fold changes from baseline in each biomarker over 12 weeks in subjects with and without disease progression over 52 weeks are shown in supplementary figure E1. There were no significant differences between fold changes in biomarkers over 12 weeks between subjects who did and did not have disease progression over 52 weeks, except for EL-NE. Adjusted mean differences in fold changes from baseline in EL-NE between subjects with *versus* without disease progression were 1.13 (95% CI 1.01–1.26) (p=0.026) at week 8 and 1.13 (95% CI 1.01–1.27) (p=0.037) at week 12.

### Performance of baseline demographic/clinical characteristics and biomarkers in classifying disease progression over 52 weeks

The proportions of subjects correctly classified as having or not having disease progression over 52 weeks in each of the models are presented in table 4. In models including only baseline demographic/clinical characteristics, 61.2–64.2% of the test set were correctly classified. In models including only baseline biomarker values, 42.6–65.6% of the test set were correctly classified. When both demographic/clinical characteristics and biomarker values were included, 50.0–64.5% of the test set were correctly classified. The factors that were selected in each of the models are summarised in supplementary table E4. When both demographic/clinical characteristics and biomarker values were included, CRP, ICAM-1, C3A and KL-6 were selected. The performance characteristics of the models are summarised in table 4.

**TABLE 4:** Performance of multivariate models for classifying subjects in the test set as having or not having disease progression over 52 weeks

	All biomarkers at baseline (n=32)	Selected biomarkers^#^ at baseline (n=61)	Demographic/clinical characteristics at baseline (n=67)	Demographic/clinical characteristics and all biomarkers at baseline (n=31)	Demographic/clinical characteristics and selected biomarkers^#^ at baseline (n=58)
**LASSO**					
Subjects correctly classified as not having disease progression	14 (43.8)	26 (42.6)	31 (46.3)	13 (41.9)	18 (31.0)
Subjects correctly classified as having disease progression	7 (21.9)	8 (13.1)	10 (14.9)	5 (16.1)	11 (19.0)
Total subjects correctly classified	21 (65.6)	34 (55.7)	41 (61.2)	18 (58.1)	29 (50.0)
Sensitivity	5.0	44.4	43.5	35.7	61.1
Specificity	77.8	60.5	70.5	76.5	45.0
Positive predictive value	63.6	32.0	43.5	55.6	33.3
Negative predictive value	66.7	72.2	70.5	59.1	72.0
**LASSO (with selection frequency ≥25%)**					
Subjects correctly classified as not having disease progression		16 (26.2)	34 (50.7)		24 (41.4)
Subjects correctly classified as having disease progression		10 (16.4)	9 (13.4)		8 (13.8)
Total subjects correctly classified		26 (42.6)	43 (64.2)		32 (55.2)
Sensitivity		55.6	39.1		44.4
Specificity		37.2	77.3		60.0
Positive predictive value		27.0	47.4		33.3
Negative predictive value		66.7	70.8		70.6
**Random forest**					
Subjects correctly classified as not having disease progression	17 (53.1)	34 (55.7)	43 (64.2)	17 (54.8)	31 (53.4)
Subjects correctly classified as having disease progression	1 (3.1)	4 (6.6)	0	3 (9.7)	1 (1.7)
Total subjects correctly classified	18 (56.3)	38 (62.3)	43 (64.2)	20 (64.5)	32 (55.2)
Sensitivity	7.1	22.2	0	21.4	5.6
Specificity	94.4	79.1	97.7	100	77.5
Positive predictive value	50.0	30.8	0	100	10.0
Negative predictive value	56.7	70.8	65.2	60.7	64.6

Data are presented as n (%) of subjects or %. LASSO: least absolute shrinkage and selection operator. **^#^**: BGM, C1M, C3A, C3M, C5M, C6M, CRP, CRPM, ICAM-1, KL-6, pro-C3, pro-C6, SP-D and VICM (see Methods for details of biomarkers) were selected as these biomarkers had an adequate number of samples for statistical testing.

## Discussion

Blood-based biomarkers predictive of short-term progression of IPF would be of clinical value. In the INMARK trial in subjects with IPF and preserved FVC, circulating levels of CRPM, C3M, CRP, KL-6 and SP-D at baseline were not significantly associated with disease progression over 52 weeks in analyses corrected for multiple comparisons. Only 50–65% of subjects in the test set were correctly classified as having or not having disease progression over 52 weeks in multivariable models that included demographic/clinical characteristics and biomarker levels at baseline.

Prior analyses of data from the INMARK trial showed that rising levels of CRPM over 12 weeks were significantly associated with disease progression over 52 weeks [12]. This was consistent with findings from the PROFILE study, which was conducted in antifibrotic drug-naive subjects with IPF who had greater impairment in FVC [3]. CRPM is generated following the degradation of CRP by MMP-1 and -8 [17], which have been shown to be elevated in patients with IPF [18, 19]. The relevance of the relationship between circulating levels of CRP and CRPM in patients with IPF remains unclear.

The heterogeneity of IPF and the complexity of the biological processes that drive fibrosis complicate the search for prognostic biomarkers, especially in the context of antifibrotic therapy. Some studies have suggested that a combination of biomarkers, or of biomarkers and clinical variables, may better identify subjects with IPF at risk of short-term progression than individual factors [4, 11, 20–24]. In a prospective cohort of 185 subjects with newly diagnosed IPF, the higher the number of neoepitopes with baseline concentrations above the median (out of C3M, C6M, pro-C3 and pro-C6), the greater the risk of disease progression or death over 6 months [24]. In a retrospective analysis of data from 118 subjects with IPF, prediction of mortality was more accurate when three circulating biomarkers (MMP-7, KL-6 and SP-A) were included in multivariate models in addition to clinical parameters (age, baseline FVC, baseline *D*_LCO_ and change in FVC over 6 months) [20]. In another study, an index based on concentrations of osteopontin, periostin, ICAM-1 and MMP-7 in combination with the GAP (gender, age and physiology) score more accurately predicted disease progression at 12 months than the GAP score alone [23]. However, in our analyses, the addition of baseline biomarker values to multivariate models did not appear to improve the proportion of subjects who were correctly classified as having disease progression over 52 weeks compared with models based on demographic/clinical variables alone. To date, no model for predicting the progression of IPF based on circulating biomarkers has been adequately validated. The challenges of identifying circulating biomarkers that are robustly associated with the progression of IPF using a targeted approach has generated interest in unbiased approaches, such as those based on machine learning or artificial intelligence, but it remains unclear whether such approaches will be more successful [25].

Strengths of our analyses include the prospective design of the INMARK trial that had a 12-week double-blind placebo-controlled period, the inclusion of patients who were naive to antifibrotic therapy and the assessment of a broad spectrum of biomarkers reflective of ECM remodelling, epithelial injury and inflammation. A limitation of our analyses is that 12 weeks might be too short a period to observe meaningful changes in FVC; thus, correlations between changes in biomarkers and changes in FVC over 12 weeks may be less informative than changes over a longer period. It should also be noted that all the subjects in the INMARK trial had preserved FVC (FVC ≥80% predicted) at baseline. It is possible that the observations relating to progression over 52 weeks were confounded by patients receiving 9 months of antifibrotic therapy. It is possible that the associations between biomarker levels and disease progression may be different in subjects with more advanced disease or in those who are treatment-naive.

In conclusion, among patients with IPF and preserved FVC in the INMARK trial, multivariate models based on a combination of demographic/clinical characteristics and biomarker levels at baseline did not provide an accurate prediction of which patients would progress. Further studies are required to inform the clinical utility of blood biomarkers in subjects with IPF.

## Supplementary material

10.1183/23120541.00335-2023.Supp1 **Please note:** supplementary material is not edited by the Editorial Office, and is uploaded as it has been supplied by the author.
Supplementary material 00335-2023.SUPPLEMENT
