# Supplementary material for: Circulating biomarkers and progression of idiopathic pulmonary fibrosis: data from the INMARK trial
Source: ERJ Open Res. 2024 Jul 22;10(4):00335-2023. doi: 10.1183/23120541.00335-2023 (PMC11261372; doi:10.1183/23120541.00335-2023)
Supplement: Supplementary file 1 [file 00335-2023.SUPPLEMENT.pdf]

Circulating biomarkers and progression of idiopathic pulmonary fibrosis: data from the INMARK trial

Online Data Supplement

Table E1. Correlations between change from baseline in each biomarker at week 4 and change from baseline in FVC % predicted at week 12.

	**Placebo**
CRPM, n	220
Spearman coefficient (95% CI)	−0.04 (−0.17, 0.09)
p-value	0.53
C1M, n	221
Spearman coefficient (95% CI)	−0.05 (−0.19, 0.08)
p-value	0.42
C3M, n	210
Spearman coefficient (95% CI)	−0.05 (−0.19, 0.08)
p-value	0.44
BGM, n	218
Spearman coefficient (95% CI)	0.11 (−0.03, 0.24)
p-value	0.11
C3A, n	221
Spearman coefficient (95% CI)	−0.01 (−0.15, 0.12)
p-value	0.83
C5M, n	219
Spearman coefficient (95% CI)	−0.08 (−0.22, 0.05)
p-value	0.21
C6M, n	201
Spearman coefficient (95% CI)	−0.04 (−0.18, 0.10)
p-value	0.54
VICM, n	221
Spearman coefficient (95% CI)	−0.14 (−0.26, 0.00)
p-value	0.044
Pro-C3, n	208
Spearman coefficient (95% CI)	0.01 (−0.13, 0.14)
p-value	0.91

Pro-C6, n	207
Spearman coefficient (95% CI)	−0.03 (−0.17, 0.11)
p-value	0.66
LOXL2, n	154
Spearman coefficient (95% CI)	−0.10 (−0.25, 0.06)
p-value	0.23
EL-NE, n	220
Spearman coefficient (95% CI)	−0.06 (−0.19, 0.07)
p-value	0.38
KL-6, n	220
Spearman coefficient (95% CI)	−0.08 (−0.21, 0.05)
p-value	0.24
SP-D, n	221
Spearman coefficient (95% CI)	−0.10 (−0.23, 0.03)
p-value	0.13
CA-125, n	112
Spearman coefficient (95% CI)	−0.07 (−0.25, 0.12)
p-value	0.48
CA19-9, n	104
Spearman coefficient (95% CI)	−0.02 (−0.21, 0.17)
p-value	0.83
CRP, n	212
Spearman coefficient (95% CI)	−0.20 (−0.32, −0.06
p-value	0.0037
ICAM-1, n	221
Spearman coefficient (95% CI)	−0.17 (−0.30, −0.04)
p-value	0.011

Table E2. Association between baseline FVC % predicted, DLco % predicted and biomarker values, and disease progression over 52 weeks.

Biomarker	Subjects with baseline biomarker value, n	Subjects with disease progression, %	Odds ratio (95% CI) for baseline FVC % predicted	Odds ratio (95% CI) for baseline DLco % predicted	Odds ratio (95% CI) for baseline biomarker level
CRPM, ng/mL	228	30.3	1.02 (1.00, 1.05)	0.99 (0.98, 1.01)	1.75 (0.99, 3.27)
C1M, ng/mL	227	30.4	1.03 (1.00, 1.05)	0.99 (0.97, 1.00)	27.7 (0.1, 11418)
C3M, ng/mL	228	30.3	1.02 (1.00. 1.05)	0.99 (0.98, 1.01)	1.91 (1.00, 3.82)*
BGM, ng/mL	226	30.5	1.02 (1.00, 1.05)	0.99 (0.98, 1.01)	1.14 (0.83, 1.60)
C3A, ng/mL	228	30.3	1.02 (1.00, 1.05)	0.99 (0.98, 1.01)	1.06 (0.65, 1.99)
C5M, ng/mL	226	30.5	1.02 (1.00, 1.05)	0.99 (0.98, 1.00)	0.89 (0.58, 1.39)
C6M, ng/mL	225	30.7	1.02 (1.00, 1.05)	0.99 (0.98, 1.01)	1.06 (0.74, 1.51)
VICM, ng/mL	228	30.3	1.02 (1.00, 1.05)	0.99 (0.98, 1.00)	0.99 (0.78, 1.25)
Pro-C3, ng/mL	220	30.9	1.03 (1.00, 1.05)	0.99 (0.98, 1.01)	1.07 (0.62, 1.85)
Pro-C6, ng/mL	218	30.7	1.03 (1.00, 1.05)	0.99 (0.98, 1.01)	0.97 (0.61, 1.53)
LOXL2, ng/mL	170	32.9	1.02 (0.99, 1.05)	0.99 (0.97, 1.00)	1.06 (0.76, 1.48)
EL-NE, ng/mL	226	30.5	1.03 (1.00, 1.05)	0.99 (0.97, 1.00)	0.81 (0.60, 1.08)
KL-6, U/mL	229	30.6	1.03 (1.00, 1.05)	1.00 (0.98, 1.01)	1.50 (1.07, 2.12)*
SP-D, ng/mL	228	30.3	1.03 (1.00, 1.05)	1.00 (0.98, 1.01)	1.49 (1.07, 2.12)*
CA-125, U/mL	154	32.5	1.04 (1.01, 1.07)	0.99 (0.98, 1.01)	0.97 (0.65, 1.47)
CA 19-9, U/mL	141	29.8	1.03 (1.00, 1.06)	1.00 (0.98, 1.01)	1.10 (0.90, 1.36)
CRP, mg/L	221	31.2	1.03 (1.00, 1.05)	1.00 (0.98, 1.01)	1.23 (1.01, 1.51)*
ICAM-1, ng/mL	228	30.3	1.03 (1.00, 1.05)	0.99 (0.98, 1.01)	1.62 (0.75, 3.55)

*p<0.05 in uncorrected analyses. p>0.05 in FDR-corrected analyses.

Table E3. Associations between baseline plus rising versus stable/falling levels of biomarkers over 12 weeks and disease progression over 52 weeks.

**Biomarker**	**Odds ratio (95% CI) for disease progression**	
	**for baseline level**	**for rising vs stable/falling levels**
CRPM, ng/mL	2.12 (1.18, 4.12)*	1.87 (1.02, 3.44)*
C1M, ng/mL	10.77 (0.02, >999.99)	1.37 (0.50, 3.76)
C3M, ng/mL	2.30 (1.10, 5.04)*	1.24 (0.63, 2.45)
BGM, ng/mL	1.14 (0.81, 1.64)	0.78 (0.41, 1.50)
C3A, ng/mL	1.17 (0.73, 2.20)	0.71 (0.36, 1.35)
C5M, ng/mL	0.89 (0.59, 1.39)	1.04 (0.58, 1.85)
C6M, ng/mL	1.28 (0.88, 1.88)	2.62 (0.80, 8.54)
VICM, ng/mL	0.98 (0.77, 1.24)	0.89 (0.47, 1.73)
Pro-C3, ng/mL	1.05 (0.61, 1.80)	1.04 (0.57, 1.86)
Pro-C6, ng/mL	0.99 (0.62, 1.58)	1.06 (0.57, 1.98)
LOXL2, ng/mL	1.05 (0.75, 1.50)	0.76 (0.38, 1.52)
EL-NE, ng/mL	0.78 (0.57, 1.07)	0.64 (0.32, 1.27)
KL-6, U/mL	1.48 (1.08, 2.04)*	1.37 (0.77, 2.49)
SP-D, ng/mL	1.44 (1.07, 1.97)*	0.92 (0.51, 1.67)
CA-125, U/mL	0.96 (0.65, 1.44)	1.62 (0.76, 3.60)
CA 19-9, U/mL	1.07 (0.88, 1.31)	NC
CRP, mg/L	1.21 (1.02, 1.45)*	NC
ICAM-1, ng/mL	1.57 (0.76, 3.31)	0.81 (0.45, 1.45)

*$p<0.05$ in uncorrected analyses. $p>0.05$ in FDR-corrected analyses. NC, not calculated (no individual slopes could be estimated in the placebo group).

Table E4. Mean coefficients or importance values for the baseline characteristics and biomarkers selected in multivariate models.

	**All biomarkers at baseline**		**Selected biomarkers* at baseline**		**Demographic/ clinical characteristics at baseline**		**Demographic/clinical characteristics and all biomarkers at baseline**		**Demographic/clinical characteristics and selected biomarkers* at baseline**	
	Variable selected	Mean coefficient or importance value	Variable selected	Mean coefficient or importance value	Variable selected	Mean coefficient or importance value	Variable selected	Mean coefficient or importance value	Variable selected	Mean coefficient or importance value
**LASSO**	No variables with selection frequency ≥25%		SP-D	0.13	Sex	0.19	No variables with selection frequency ≥25%		C3M	0.13
			C3M	0.09	Race	0.12			BMI	−0.22
					BMI	−0.10			Sex	0.12
									CRP	0.15
									SP-D	0.09
									ICAM-1	0.08
									FVC % predicted	0.10
**Random forest**	CRP	10.0	SP-D	10.0	Sex	10.0	CRP	10.0	C3M	10.0
	C3M	6.7	CRPM	8.6	Race	4.5	ICAM-1	2.7	ICAM-1	9.3
	SP-D	5.6	KL-6	3.9	FVC % predicted	4.3	C3A	2.6	FVC % predicted	8.9
	C6M	4.8	C3M	3.4			KL-6	2.6	Sex	6.0

	VICM	4.7	C3A	3.3				CRPM	5.3
	BGM	2.8	C6M	2.6				BMI	5.3
	Pro-C3	2.6						KL-6	3.9
								CRP	3.7
								Race	2.7

*BGM, C1M, C3A, C3M, C5M, C6M, CRP, CRPM, ICAM-1, KL-6, pro-C3, pro-C6, SP-D and VICM were selected as these biomarkers had an adequate number of samples for statistical testing. Variables had selection frequency ≥25% in the LASSO model with stability selection or importance values ≥2.5 in the random forest model. Importance values were standardized to a range from 0 to 10, *i.e.,* displayed according to their position within this range.

Figure E1. Fold change in each biomarker over 12 weeks in subjects with and without disease progression over 52 weeks.

CRPM

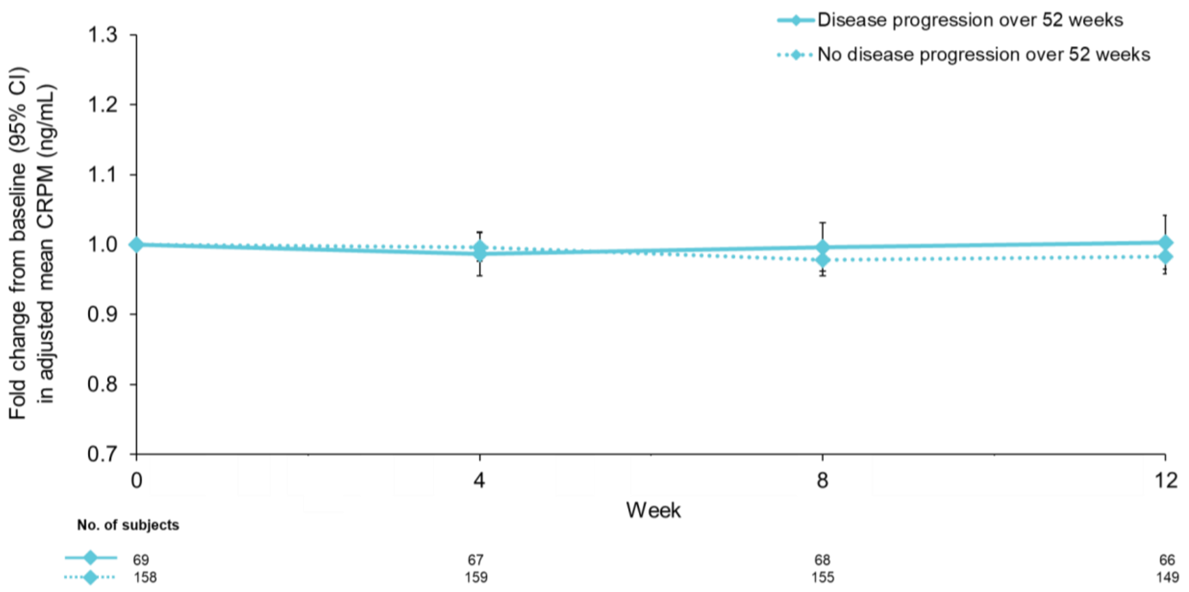

C3M

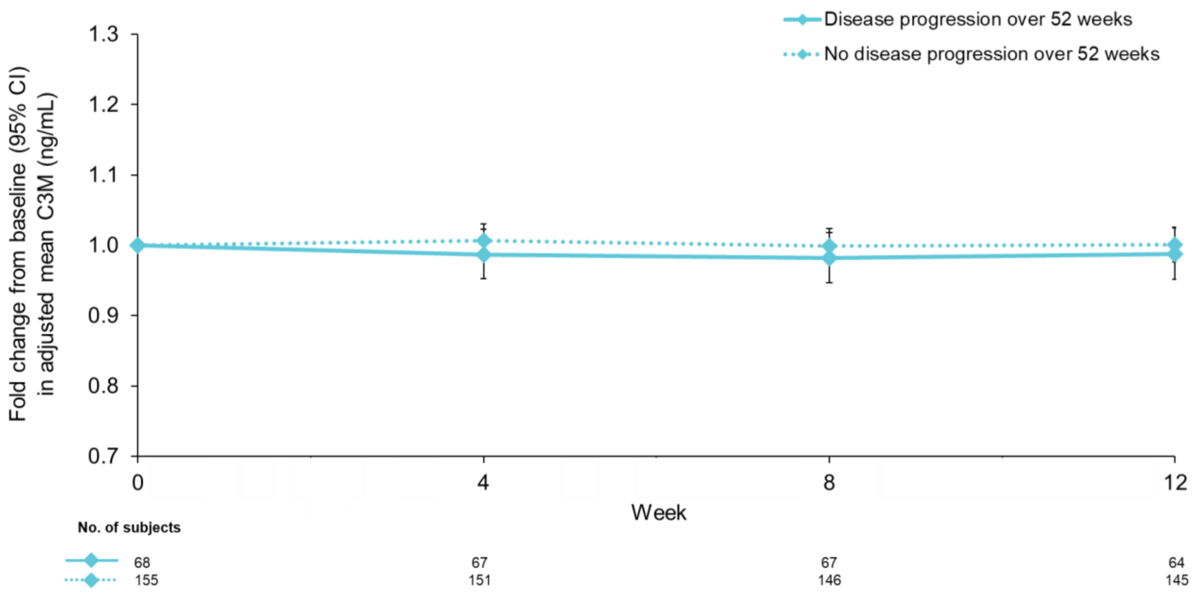

BGM

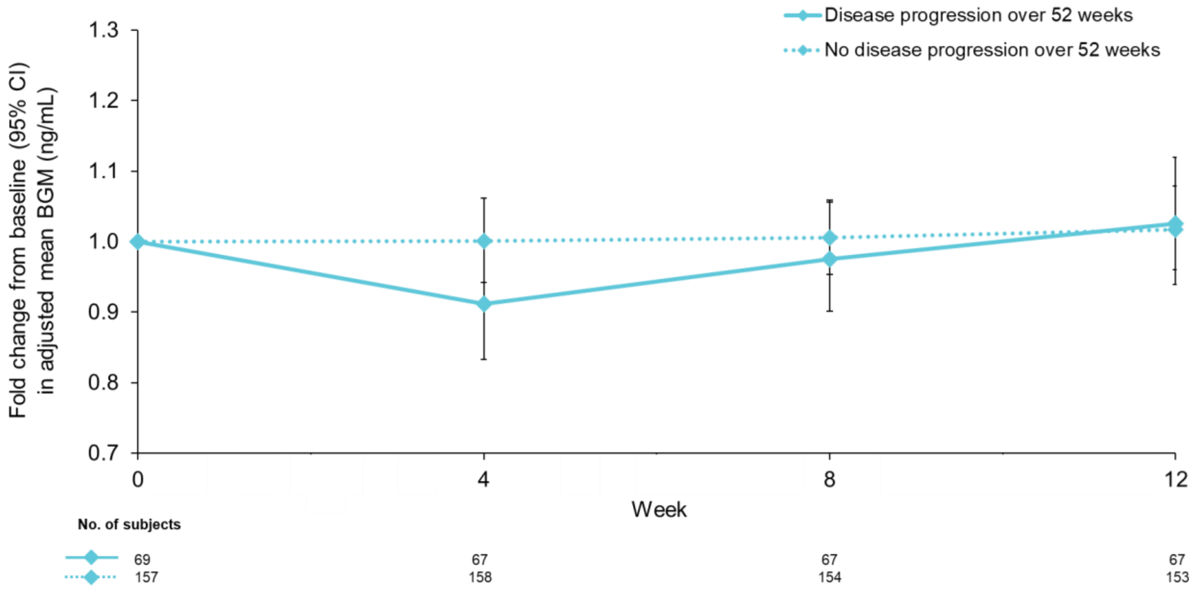
Disease progression over 52 weeks
No disease progression over 52 weeks
Fold change from baseline (95% CI) in adjusted mean BGM (ng/mL)
1.3
1.2
1.1
1.0
0.9
0.8
0.7
0
4
8
12
Week
No. of subjects
69 67 67 67
157 158 154 153

C3A

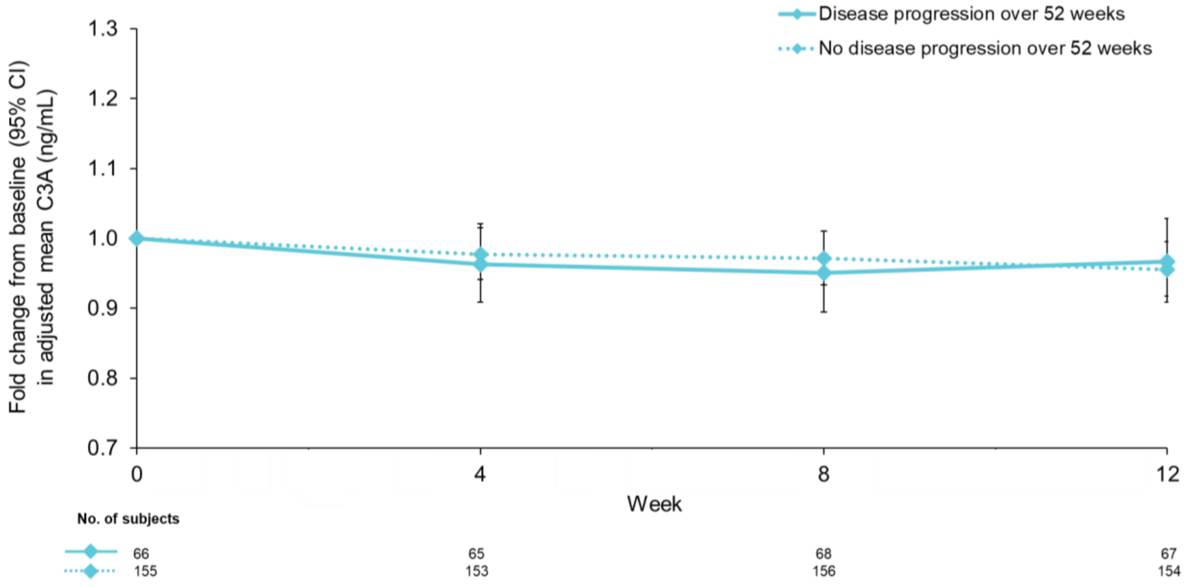
Disease progression over 52 weeks
No disease progression over 52 weeks
Fold change from baseline (95% CI) in adjusted mean C3A (ng/mL)
1.3
1.2
1.1
1.0
0.9
0.8
0.7
0
4
8
12
Week
No. of subjects
66 65 68 67
155 153 156 154

C5M

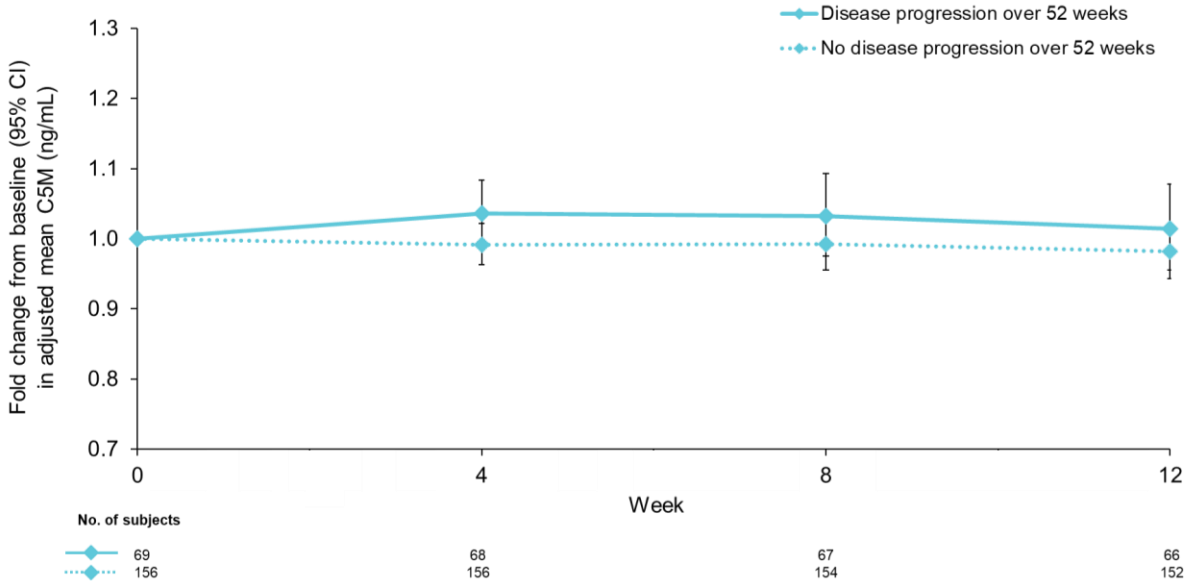
Disease progression over 52 weeks
No disease progression over 52 weeks
Fold change from baseline (95% CI) in adjusted mean C5M (ng/mL)
1.3
1.2
1.1
1.0
0.9
0.8
0.7
0
4
8
12
Week
No. of subjects
69 68 67 66
156 156 154 152

C6M

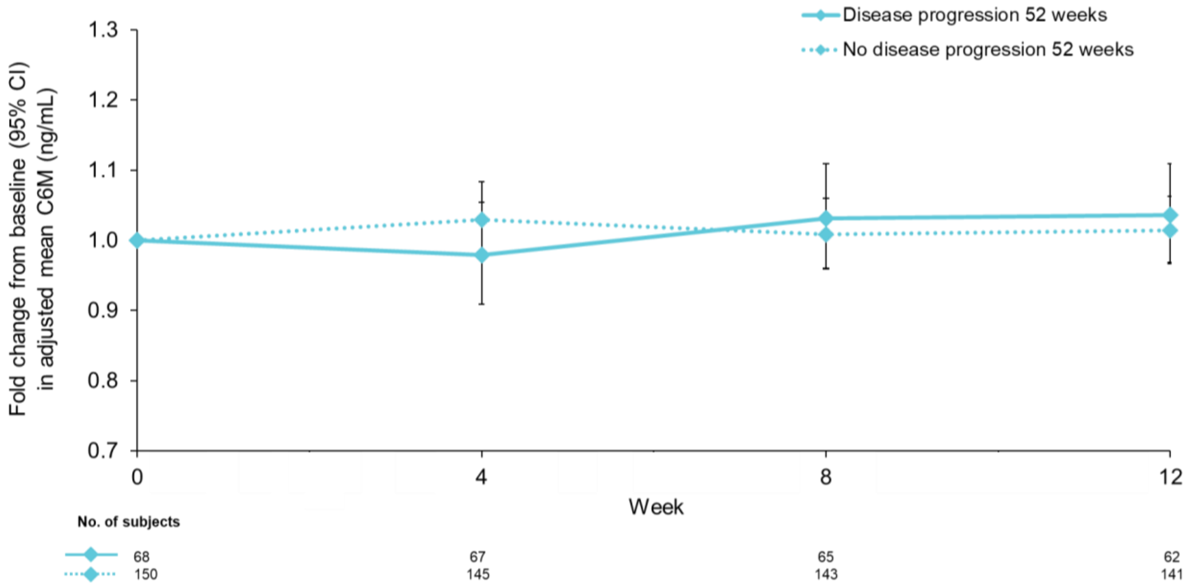
Disease progression 52 weeks
No disease progression 52 weeks
Fold change from baseline (95% CI) in adjusted mean C6M (ng/mL)
1.3
1.2
1.1
1.0
0.9
0.8
0.7
0
4
8
12
Week
No. of subjects
68 67 65 62
150 145 143 141

VICM

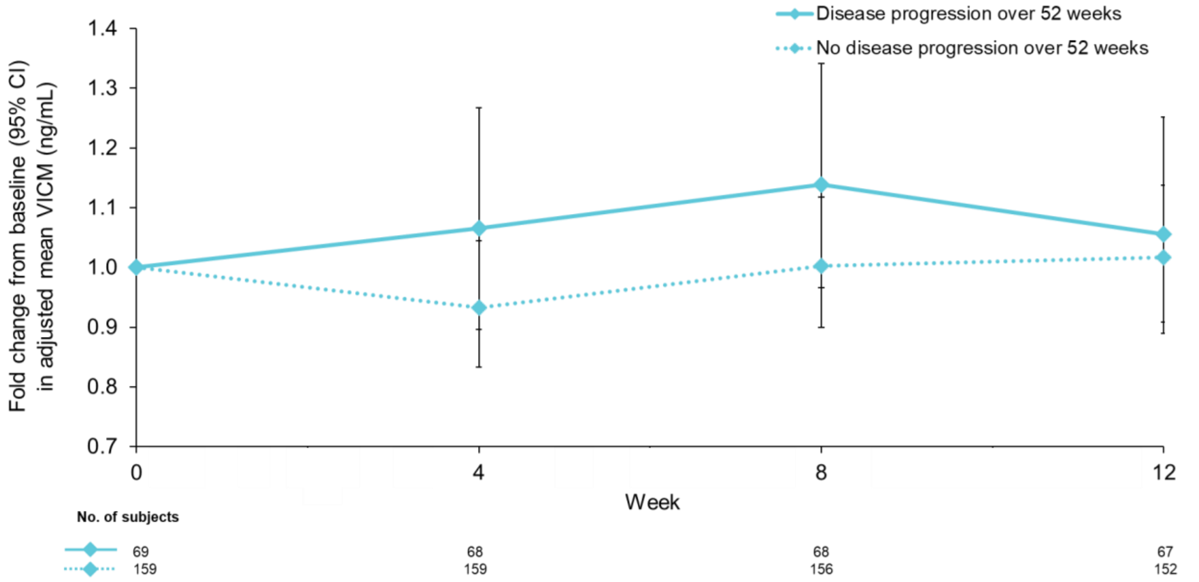
Disease progression over 52 weeks
No disease progression over 52 weeks
Fold change from baseline (95% CI) in adjusted mean VICM (ng/mL)
1.4
1.3
1.2
1.1
1.0
0.9
0.8
0.7
0
4
8
12
Week
No. of subjects
69
159
68
159
68
156
67
152

Pro-C3

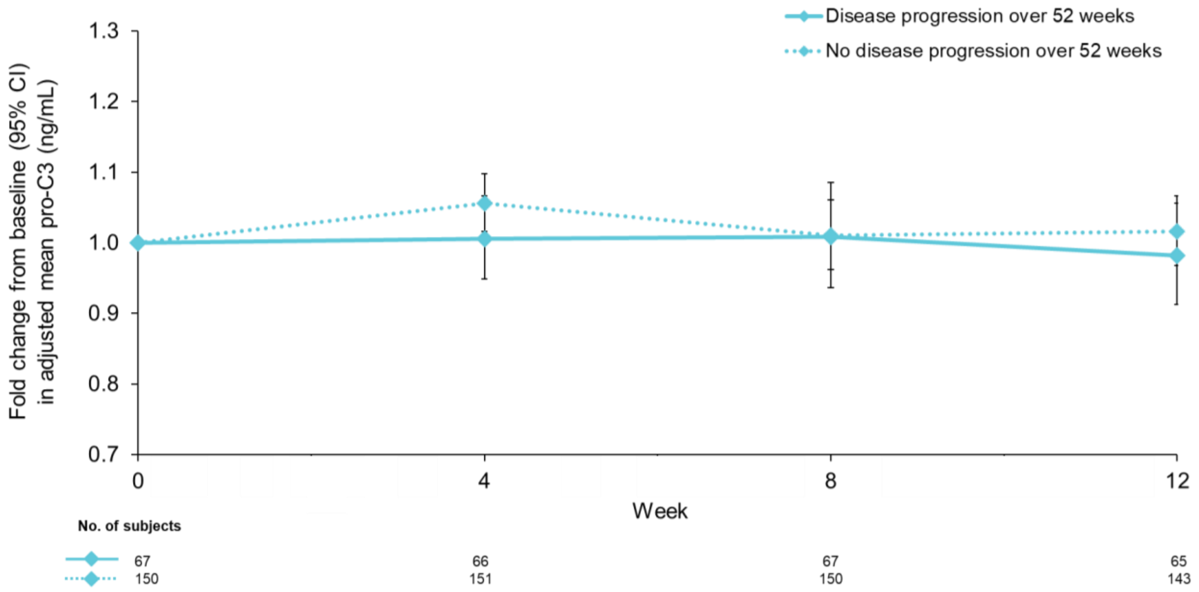
Disease progression over 52 weeks
No disease progression over 52 weeks
Fold change from baseline (95% CI) in adjusted mean pro-C3 (ng/mL)
1.3
1.2
1.1
1.0
0.9
0.8
0.7
0
4
8
12
Week
No. of subjects
67
150
66
151
67
150
65
143

Pro-C6

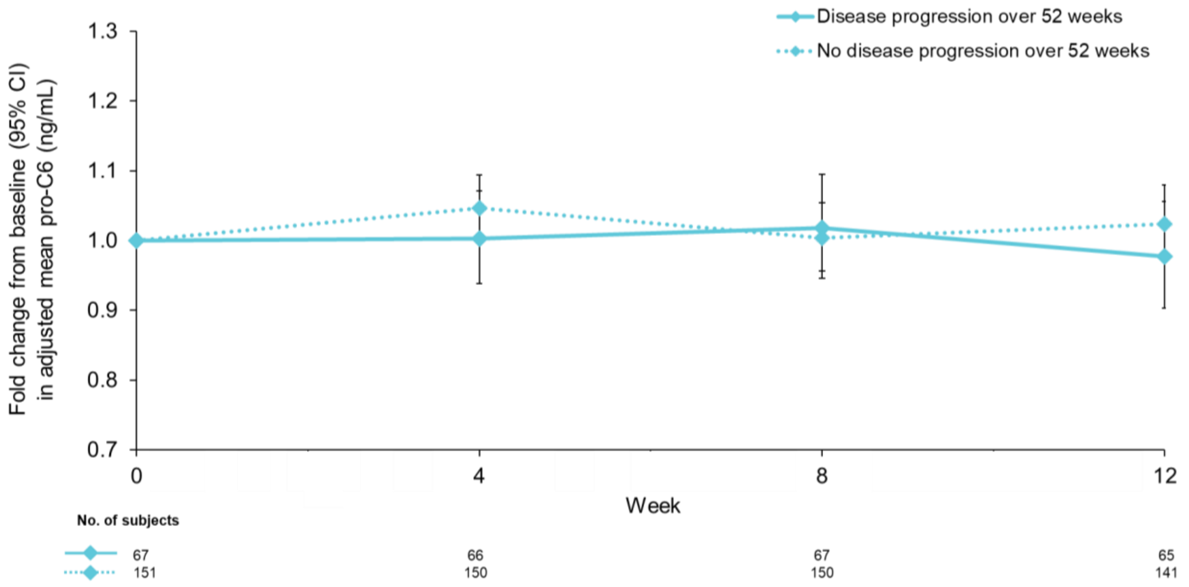

Disease progression over 52 weeks
No disease progression over 52 weeks
Fold change from baseline (95% CI) in adjusted mean pro-C6 (ng/mL)
1.3
1.2
1.1
1.0
0.9
0.8
0.7
0
4
8
12
Week
No. of subjects
67
151
66
150
67
150
65
141

LOXL2

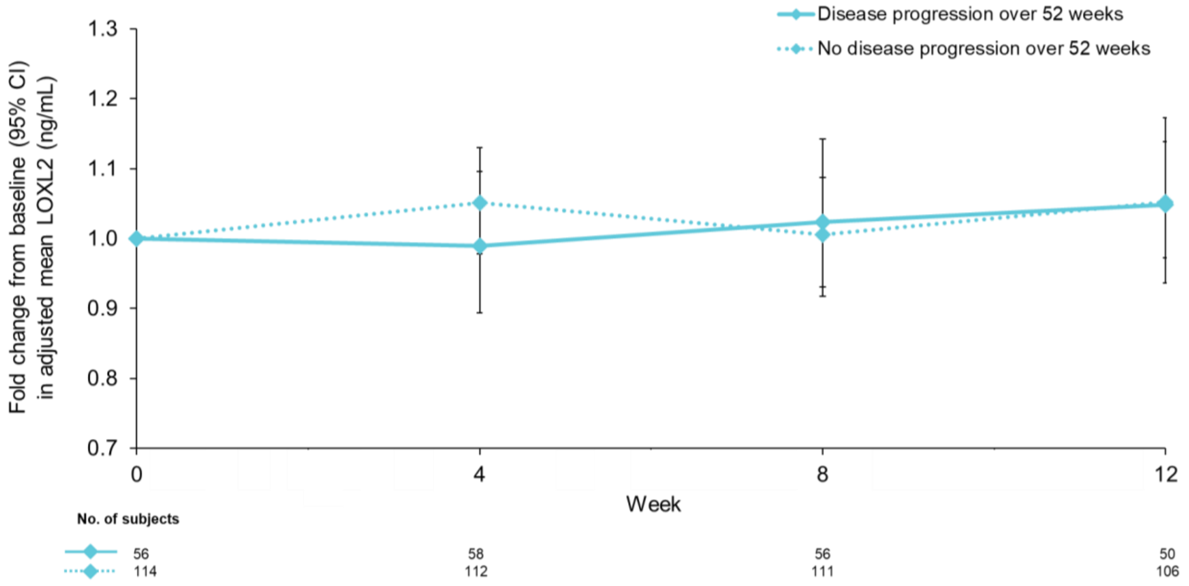

Disease progression over 52 weeks
No disease progression over 52 weeks
Fold change from baseline (95% CI) in adjusted mean LOXL2 (ng/mL)
1.3
1.2
1.1
1.0
0.9
0.8
0.7
0
4
8
12
Week
No. of subjects
56
114
58
112
56
111
50
106

EL-NE

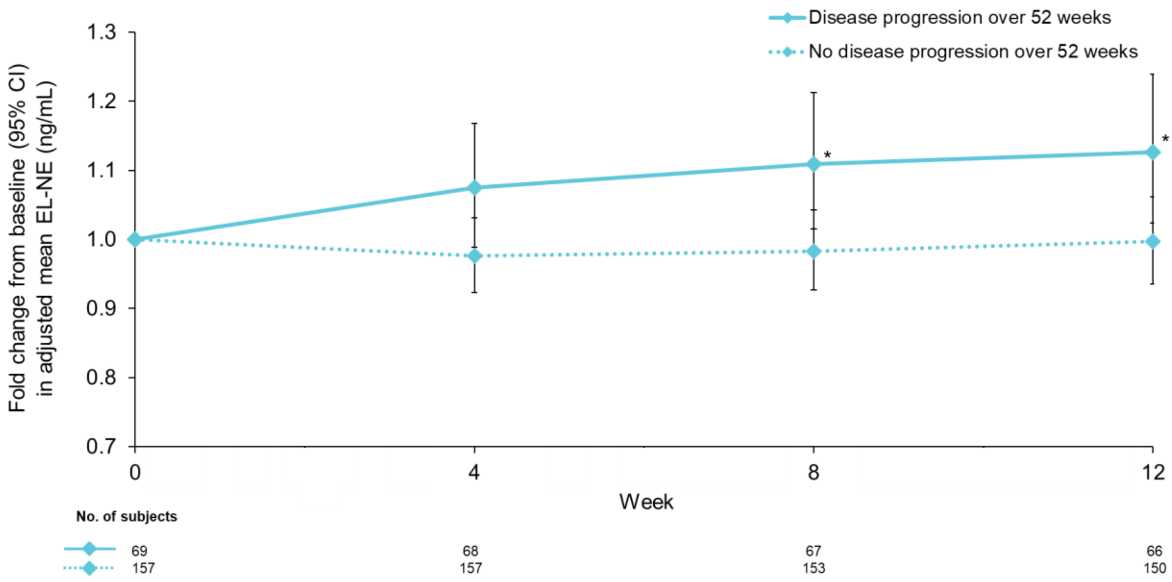

*p<0.05 for the comparison with no disease progression.

KL-6

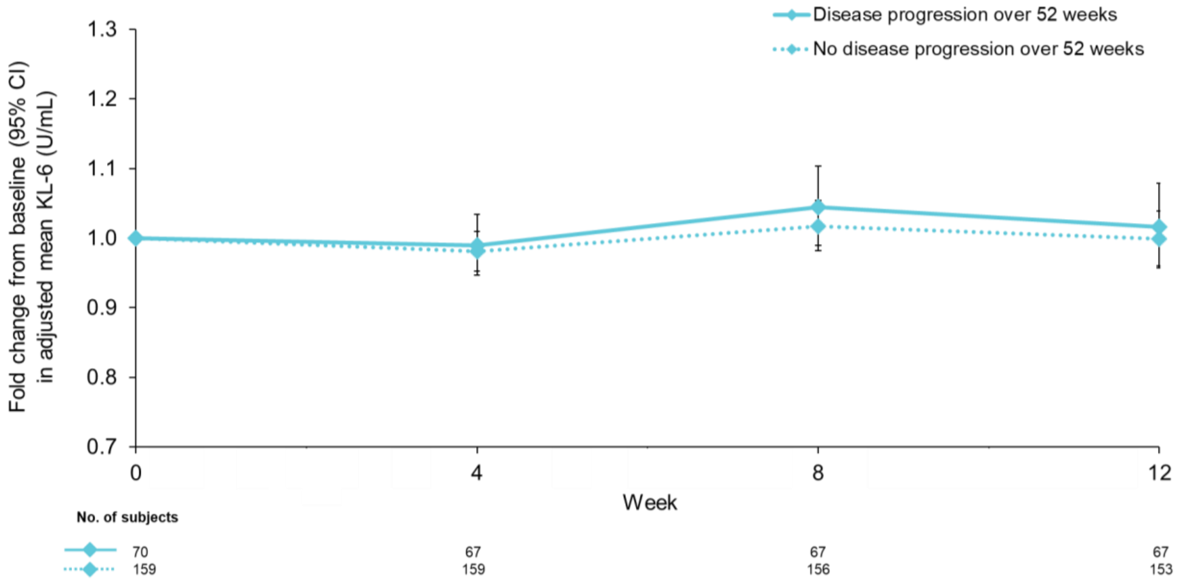

SP-D

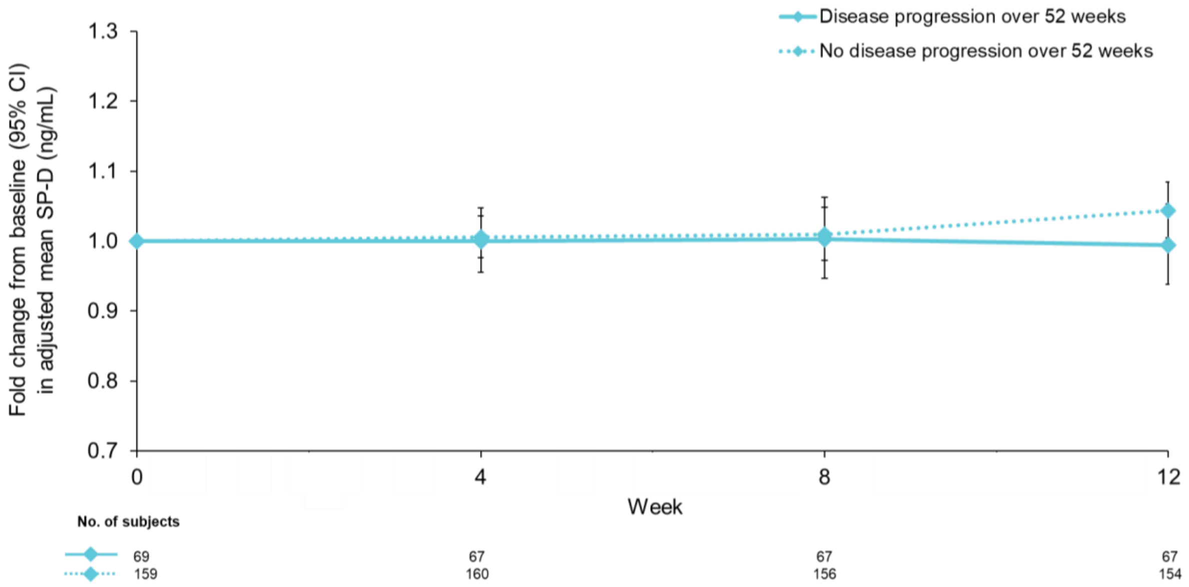
Disease progression over 52 weeks
No disease progression over 52 weeks
Fold change from baseline (95% CI) in adjusted mean SP-D (ng/mL)
1.3
1.2
1.1
1.0
0.9
0.8
0.7
0
4
8
12
Week
No. of subjects
69 67 67 67
159 160 156 154

CA-125

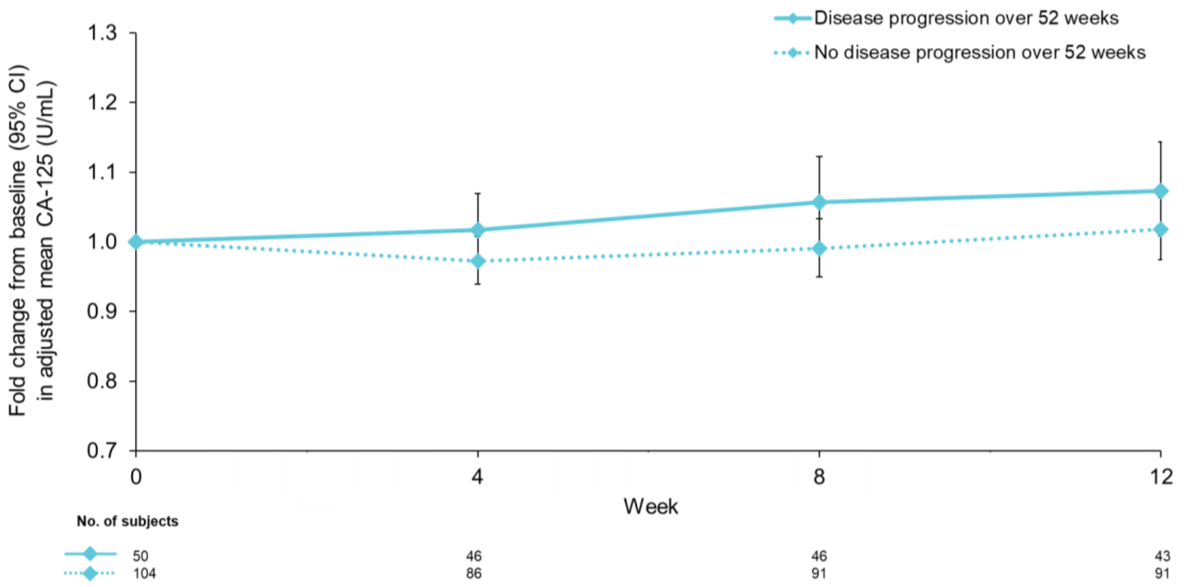
Disease progression over 52 weeks
No disease progression over 52 weeks
Fold change from baseline (95% CI) in adjusted mean CA-125 (U/mL)
1.3
1.2
1.1
1.0
0.9
0.8
0.7
0
4
8
12
Week
No. of subjects
50 46 46 43
104 86 91 91

CA19-9

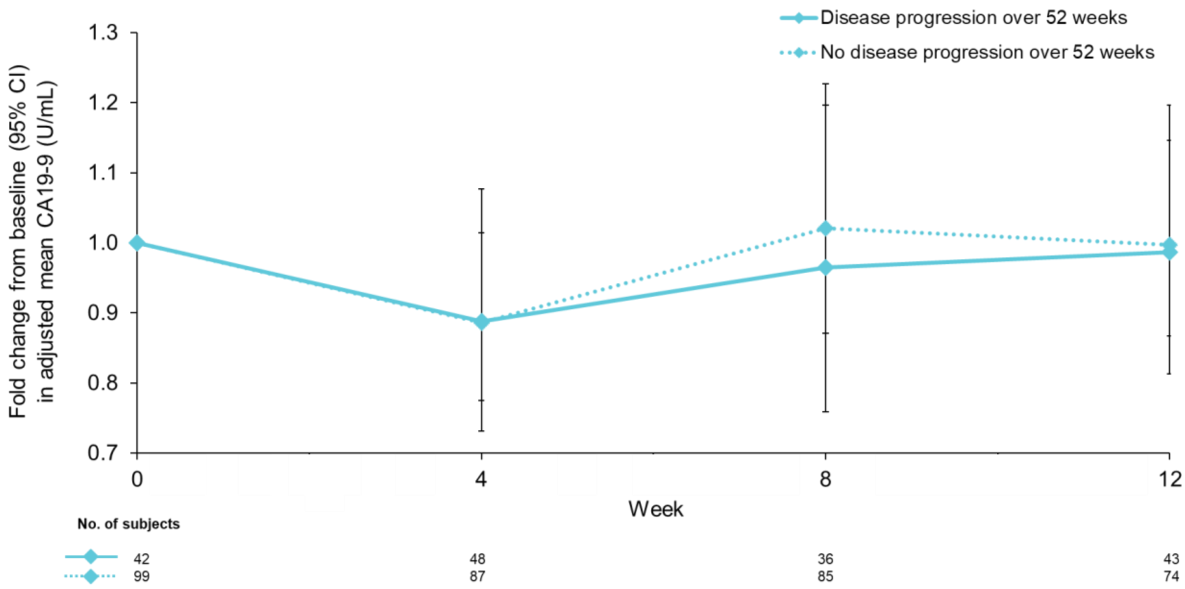
Disease progression over 52 weeks
No disease progression over 52 weeks
Fold change from baseline (95% CI) in adjusted mean CA19-9 (U/mL)
1.3
1.2
1.1
1.0
0.9
0.8
0.7
0
4
8
12
Week
No. of subjects
42
99
48
87
36
85
43
74

CRP

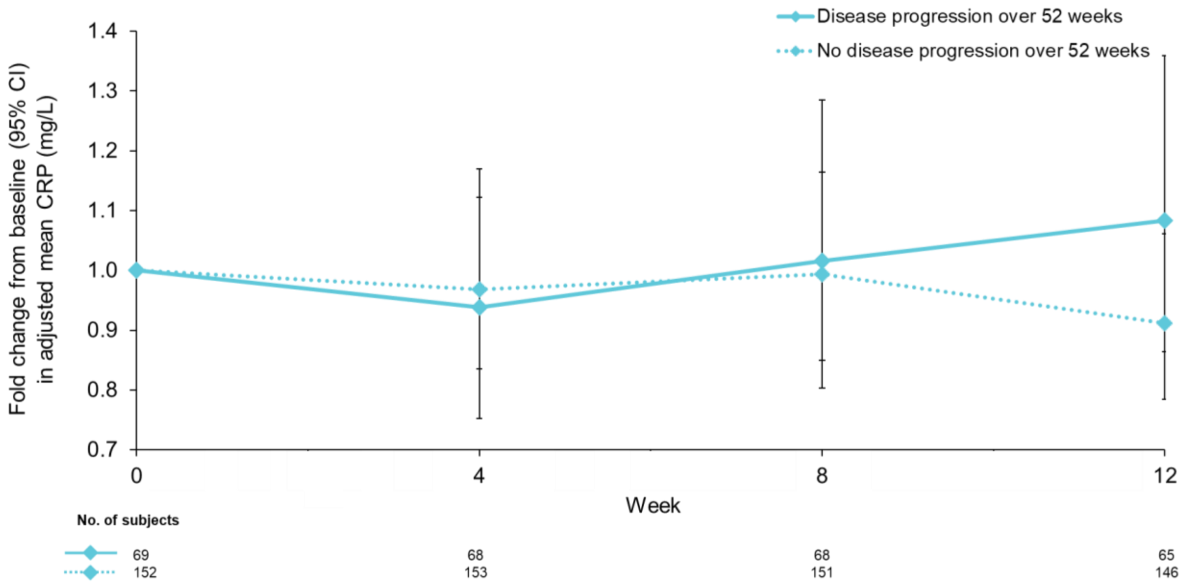
Disease progression over 52 weeks
No disease progression over 52 weeks
Fold change from baseline (95% CI) in adjusted mean CRP (mg/L)
1.4
1.3
1.2
1.1
1.0
0.9
0.8
0.7
0
4
8
12
Week
No. of subjects
69
152
68
153
68
151
65
146

ICAM-1

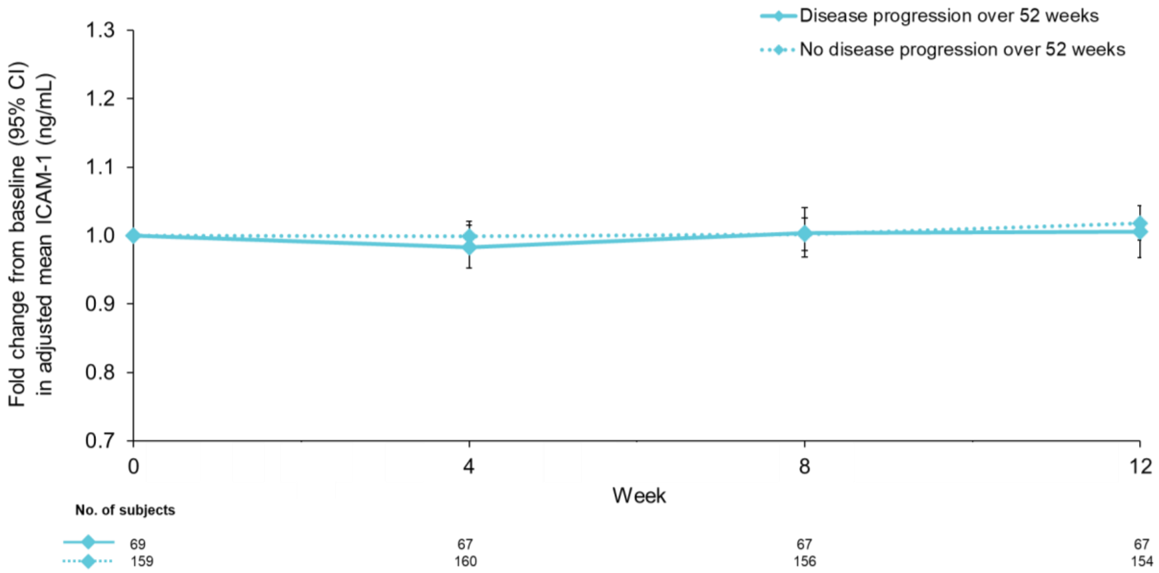

Disease progression over 52 weeks
No disease progression over 52 weeks
Fold change from baseline (95% CI) in adjusted mean ICAM-1 (ng/mL)
1.3
1.2
1.1
1.0
0.9
0.8
0.7
0
4
8
12
Week
No. of subjects
69
67
67
67
159
160
156
154
